# A Comparative Study of Enantioseparations of *N*^α^-Fmoc Proteinogenic Amino Acids on *Quinine*-Based Zwitterionic and Anion Exchanger-Type Chiral Stationary Phases under Hydro-Organic Liquid and Subcritical Fluid Chromatographic Conditions

**DOI:** 10.3390/molecules21111579

**Published:** 2016-11-22

**Authors:** Gyula Lajkó, Nóra Grecsó, Gábor Tóth, Ferenc Fülöp, Wolfgang Lindner, Antal Péter, István Ilisz

**Affiliations:** 1 Department of Inorganic and Analytical Chemistry, University of Szeged, Dóm tér 7, H-6720 Szeged, Hungary; gyula.lajko@pharm.u-szeged.hu (G.L.); grecso.nora@pharm.u-szeged.hu (N.G.); apeter@chem.u-szeged.hu (A.P.);; 2 Institute of Pharmaceutical Chemistry, University of Szeged, Eötvös u. 6, H-6720 Szeged, Hungary; ferenc.fulop@pharm.u-szeged.hu; 3 Institute of Medical Chemistry, University of Szeged, Dóm tér 8, H-6720 Szeged, Hungary; toth.gabor@med.u-szeged.hu; 4 Department of Analytical Chemistry, University of Vienna, Währinger Strasse 38, 1090 Vienna, Austria; wolfgang.lindner@univie.ac.at

**Keywords:** enantiomer separation, hydro-organic liquid chromatography, subcritical fluid chromatography, zwitterionic chiral stationary phase, weak anion-exchange chiral stationary phase, *N*^α^-Fmoc-protected proteinogenic amino acids, temperature effect

## Abstract

The focus of this contribution is a comparative investigation of enantioseparations of 19 *N*^α^-Fmoc proteinogenic amino acids on *Quinine*-based zwitterionic and anion-exchanger type chiral stationary phases employing hydro-organic and polar-ionic liquid and subcritical fluid chromatographic conditions. Effects of mobile phase composition (including additives, e.g., water, basis and acids) and nature of chiral selectors on the chromatographic performances were studied at different chromatographic modes. Thermodynamic parameters of the temperature dependent enantioseparation results were calculated in the temperature range 5–50 °C applying plots of lnα versus 1/*T*. The differences in standard enthalpy and standard entropy for a given pair of enantiomers were calculated and served as a basis for comparisons. Elution sequence in all cases was determined, where a general rule could be observed, both in liquid and subcritical fluid chromatographic mode the d-enantiomers eluted before the L ones. In both modes, the principles of ion exchange chromatography apply.

## 1. Introduction

The stereochemical (chiral) purity of biologically active compounds became a strongly emerging issue in the last decades, which eventually impacted the synthetic concepts of drugs and intermediates but also the related analytics. The determination of the enantiomeric excess (*e.e.*) and stereochemical purity became essential in all aspects in pharmaceutical industry and in life sciences. Stereoisomeric impurities may have unwanted toxicological, pharmacological, or other side-effects, therefore their presence must be analytically controlled [1,2]. In addition to problems in drug discovery, the *e.e.* issues are also of importance for the successful chemical synthesis of different biomolecules including therapeutic peptides and proteins [3]. Especially for multistep syntheses concepts involving chiral synthons their rigorous chemical and stereochemical purity control is highly demanded and often a challenging task [4].

As a great variety of *N*^α^-protected amino acids serves as building blocks (chiral synthons) for modern peptide syntheses, there is a great need to control and analyze them with high sensitivity. Gas- and liquid-chromatographic methods besides capillary electrophoresis are one of the best choices for discrimination of chiral compounds. At early stage, the so-called “indirect” concept of chiral analysis was favored applying chiral derivatizing agents (CDAs), but was soon outperformed by the so-called “direct” enantioseparation techniques using chiral mobile phase additives or chiral stationary phases (CSPs). In the context of this study, a fair number of papers have already dealt with the enantioseparation of free- and *N*^α^-protected proteinogenic and unusual amino acids, as summarized in several review papers [5,6,7,8,9,10,11,12,13,14].

In the last decade, the supercritical fluid chromatography (SFC) with mobile phases of liquid carbon dioxide at super- or subcritical state containing co-solvents (most often MeOH) became a powerful chromatographic technique for the enantioseparation of chemically and biologically important molecules. As the polarity of liquid CO_2_ is claimed to be comparable to n-hexane, to increase the polarity of the mobile phases (MPs), polar organic modifiers are required to ensure solubility of analytes, and also to enhance the elution strength of the MP. For variation of the overall solvent strength, many polar solvents such as methanol, acetonitrile, THF, etc. and also additives such as organic bases and acids and/or organic salts can be mixed with liquid CO_2_ at high pressure, thus the mobile phase polarity can easily be adjusted [15,16,17,18]. Most of the CSPs regularly used for enantiomer separations in HPLC can also be operated under SFC conditions, thus, via a comparative study, valuable information can be obtained.

The *Cinchona* alkaloid-based zwitterionic CSPs were already successfully utilized for the enantiomeric separation of chiral acids, amines, amino acids and small peptides in polar-ionic (PI) and reversed-phase (RP) modes by HPLC [19,20]. Enantioseparations of some *N*^α^-protected proteinogenic amino acids were carried out by these zwitterionic CSPs employing also SFC conditions [21], while weak anion-exchange type [22] and strong cation-exchange type CSPs and their analogs have been applied for the enantioseparation of various chiral acids and chiral basic compounds [23,24].

Mechanistically, enantioseparation is usually achieved through the formation of transitional diastereomeric complex, formed between the analytes (selectands, SAs) and the chiral selector (SO), which get modulated via the mobile phase components. In most cases, the stereoselective interactions are also sensitive to temperature [25,26,27,28,29]. Accordingly, in chiral separations the column temperature has often been optimized and kept well controlled [25,26,27,28,29,30,31,32,33,34].

The difference in the change in standard enthalpy *Δ*(*ΔH°*) and entropy *Δ*(*ΔS°*) for enantiomers can be determined from the van’t Hoff equation [29]:

(1) $$ln\;\alpha =-\frac{\Delta \left(\Delta H{}^{\circ}\right)}{RT}+\frac{\Delta \left(\Delta S{}^{\circ}\right)}{R}$$

where *R* is the gas constant, *T* is temperature in Kelvin and α is the selectivity factor. If a linear van’t Hoff plot is obtained, a plot of R ln α versus 1/*T* has a slope of −*Δ*(*ΔH*°) and an intercept of *Δ*(*ΔS*°). In the present paper, the achiral and chiral contributions to the retention [30,31,32] are not differentiated, i.e., the classical van’t Hoff approach assuming only one type of interaction sites was used. A more sophisticated interpretation would be based on the discrimination of the contributions of the enantioselective and non-selective sites [28,30,31,32]; however, this approach has its own limitations.

**Figure 1:**
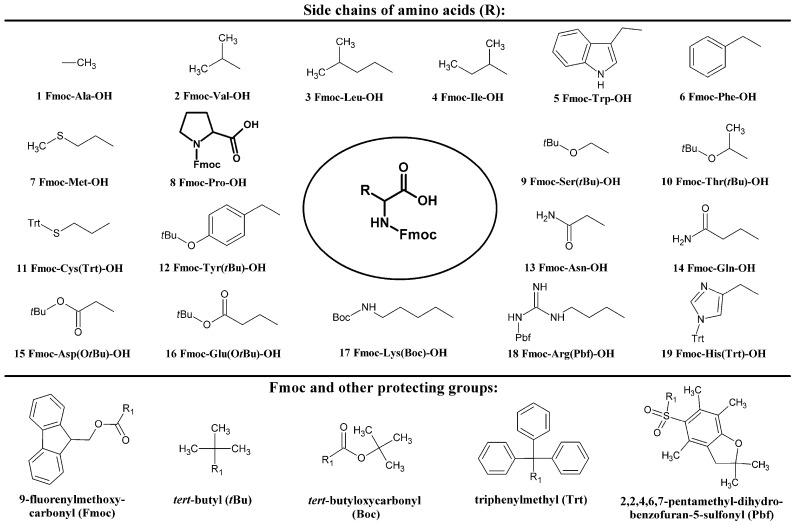
Structure of analytes.

**Figure 2:**
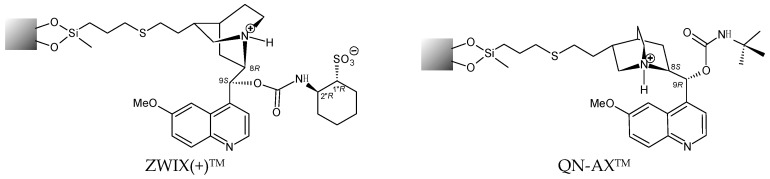
Structure of Chiralpak ZWIX(+)™ and QN-AX™ CSPs. Grey ball: represents the silica matrix.

In this work, we present results obtained on the enantioseparation of *N*^α^-Fmoc protected proteinogenic amino acids (Figure 1) on *Cinchona* alkaloid *quinine* (QN)-based zwitterionic and weak anion exchanger-type chiral stationary phases (Figure 2) in hydro-organic (HO) and polar-ionic (PI) mode by HPLC, and by SFC. The influence of mobile phase composition, nature of chiral selector and the effect of temperature on the chromatographic performance and enantioselectivity are described in a comparative fashion. In addition, a detailed study on the effect of additives (water, acids, bases or salts) was undertaken. The elution sequence was determined in all cases, as it reflects the molecular recognition principles.

## 2. Results

Of the nineteen *N*^α^-Fmoc proteinogenic amino acids (Figure 1), five compounds, Fmoc-Asp(O*t*Bu)-OH, Fmoc-Lys(Boc)-OH, and Fmoc-Leu-OH, and two aromatic side chains containing Fmoc-Phe-OH and Fmoc-Tyr(*t*Bu)-OH), were chosen as model analytes with an overall acidic character to discuss diverse method related effects.

### 2.1. Separation of N^α^-Fmoc Proteinogenic Amino Acids on Quinine-Based CSPs under Liquid Chromatographic Conditions

#### 2.1.1. Effect of Bulk Solvent Composition

As reported previously, the *Cinchona* alkaloid-based zwitterionic chiral ion-exchangers can be used as enantioselective cation- and anion-exchangers, as well as ampholytic double ion-exchangers in retaining ampholytes via zwitterionic retention mechanisms in polar-ionic mode (PIM) in combination with a mixture of MeOH and MeCN as polar solvents [19,20]. MeOH may suppress *H*-bonding interactions, while MeCN may support ionic interactions, but interferes with aromatic (π–π) interactions.

**Figure 3:**
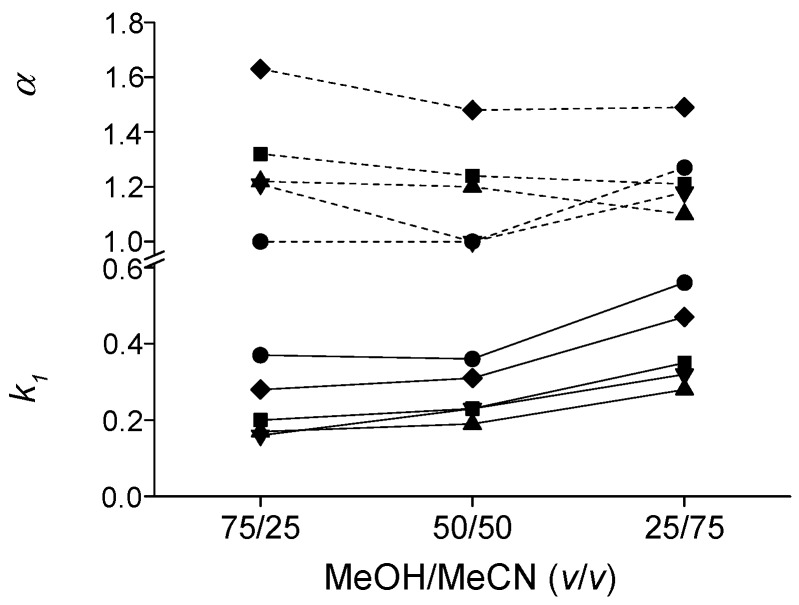
Effects of the bulk solvent composition on chromatographic parameters, *k*_1_ and *α.* Chromatographic conditions: column, ZWIX(+)™; mobile phase, MeOH/MeCN (70/25, 50/50 and 25/75 *v/v*) all containing 30 mM TEA and 60 mM FA; flow rate, 0.6 mL·min^−1^; detection, 262 nm; symbols: ■ for Fmoc-Asp(O*t*Bu)-OH; ◆ for Fmoc-Phe-OH; ▲ for Fmoc-Leu-OH; ▼ for Fmoc-Lys(Boc)-OH; and ⚫ for Fmoc-Tyr(*t*Bu)-OH.

For investigation of the effects of bulk solvent composition, enantioseparations of the five model compounds were achieved on ZWIX(+)™ in MeOH/MeCN (75/25, 50/50 and 25/75 *v/v*) mobile phases containing 30 mM TEA and 60 mM FA (Figure 3). Applying mobile phases containing MeOH/MeCN, very low *k* values were obtained: *k_1_* varied between 0.16 and 0.56, and *k_1_* increased with increasing MeCN content. The higher content of MeCN supported the ionic interactions, which resulted in longer retention. Due to the *N*^α^-Fmoc protection (and Boc protection), the basicity of the amino group(s) is suppressed and therefore a double ion-pairing process with the zwitterionic CSP (as it is the case for free amino acids) is not possible, resulting in rather low retentions. Only an anion exchange retention mechanism is in place. However, at least partial resolutions could be obtained in many cases, while Fmoc-Phe-OH and Fmoc-Tyr(*t*Bu)-OH could be baseline resolved under these conditions.

To improve the separation performances on ZWIX(+)™, besides MeOH/MeCN as bulk solvent, several PI and HO mobile phase combinations such as MeOH/THF, MeCN/THF, MeOH/MeCN/THF, MeOH/MeCN/H_2_O, MeCN/H_2_O and MeOH/H_2_O all containing an organic acid and a base additive at a ratio of 2:1 were applied. Based on these experiments (data not shown), it can be stated that the presence of H_2_O in the mobile phase has a slight positive effect on the enantioselectivity, while other mobile phase combinations were found to be insufficient regarding the chiral discrimination. Applying an eluent containing 1–2 *v*% H_2_O was advantageous for the enantioseparations, while a further increase of the H_2_O content resulted in a decrease in the chromatographic parameters. In H_2_O/MeOH (1/99 *v/v*) mobile phases containing 30 mM TEA and 60 mM FA, *k_1_* values were slightly higher compared to the results obtained with MeOH/MeCN mobile phases, while *α* values increased by about 30% and most of the Fmoc-protected amino acids exhibited partial or baseline separation (exceptions were Fmoc-Pro-OH, Fmoc-Thr(*t*Bu)-OH and Fmoc-Tyr(*t*Bu)-OH) (Table 1).

In the case of the studied model compounds, the primary interaction, decisive in regard to retention, is the ionic interaction between the cationic sites of the SO and anionic site of the SA, with additional intermolecular SO–SA interactions responsible for chiral discrimination. Because of the Fmoc-protection, the cationic site of the free amino acid is transferred to a slightly acidic hydrogen donating site shifting the double ion pairing towards a cation exchanger (mono ion pairing) concept. This is the possible reason why the *Cinchona* alkaloid-based zwitterionic CSP exhibited somewhat poor separation ability under PIM or HO mobile phase conditions. The dedicated weak anion-exchanger type CSP, QN-AX (Figure 2) differs structurally from the zwitterionic-based selectors, as it possesses a neutral *t*-butylcarbamoylated moiety instead of the negatively charged cyclohexylsulfonyl site (Figure 2). However, the positively charged site for the anion exchanger remains the same in the selector motifs whereas the ZWIX phases contain the negatively charged site (the sulfonic acid group), which acts repulsive for acidic analytes. Overall, the QN-AX phase is, therefore, better suited for the retention and resolution of acidic (negatively charged) analytes.

For the investigation of bulk solvent composition on QN-AX™ the chromatographic separations were carried out in MeOH/MeCN (80/20, 70/30 and 50/50 *v/v*) mobile phases containing 30 mM TEA and 60 mM FA. The *k*_1_ values ranged between 1.4 and 4.2, *α* between 1.4 and 1.9 and *R*_S_ between 6.2 and 10.0 (data not shown). *k*_1_, *α* and *R_S_* values slightly decreased with increasing MeCN content in contrast to the values observed on zwitterionic SOs where *k*_1_, *α* and *R_S_* slightly increased with increasing MeCN content. However, similar behavior was found in the case of the α-amino acids, where higher MeCN content usually leads to decreased enantioselectivity and resolution [35]. *k*_1_, *α* and *R_S_* values were much higher than the ones obtained on ZWIX(+)™ at HO mobile phase and in all cases baseline separations were observed, *R_S_* >> 1.5 (the only exception was Fmoc-Thr(*t*Bu)-OH, Table 1).

#### 2.1.2. Effect of Base and Acid Additives

The optimization of the nature and concentration of the various acid and base additives applied in the mobile phase is of importance when applying *Cinchona* alkaloid-based CSPs. Earlier investigations showed that in anion exchange mode acid additives act as counter-ions, while bases as co-ions [35,36,37]; the co- and counter-ions are able to change the solvation effects of the SO and SA moieties and interaction sites thus influencing also the eluent properties.

**Figure 4:**
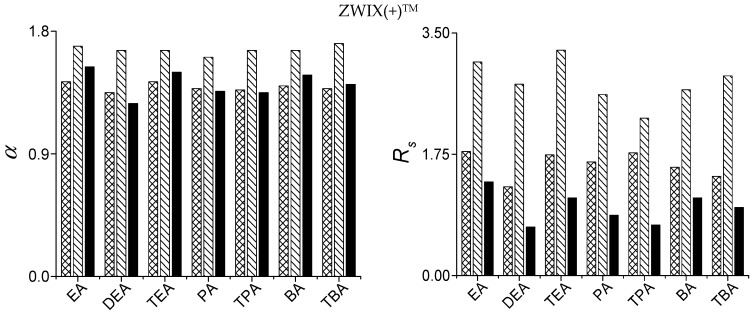
Effects of the natures of base additives on the chromatographic parameters of *α* and *R_S_* of Fmoc-Asp(O*t*Bu)-OH, Fmoc-Lys(Boc)-OH and Fmoc-Phe-OH. Chromatographic conditions: columns, ZWIX(+)™; mobile phase, H_2_O/MeOH (1.0/99.0 *v/v*) containing 3.75 mM base and 7.5 mM AcOH; flow rate, 0.6 mL·min^−1^; detection, 262 nm. Symbols: 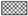 for Asp(O*t*Bu)-OH; 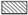 for Fmoc-Lys(Boc)-OH; and 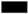 Fmoc-Phe-OH.

Effects of acid and base additives were investigated on ZWIX(+)™ column for all five selected SAs at a mobile phase composition H_2_O/MeOH (1/99 *v/v*) containing EA, DEA, TEA, PA, TPA, BA or TBA as base, and FA or AcOH as acid keeping the acid-to-base ratio of 2:1. Acid excess in the mobile phase ensured that the bases (amines) were present in their ammonium-ion forms. The amines differed in the degree and nature of their alkyl substitution, while the acids differed by one methyl group. In contrast with the results obtained in PIM for the free α- and β-amino acids where *k*_1_ slightly increased as the degree of ethyl or propyl substitution on the *N* atom increased [38,39,40], in the present study only slight differences in *k*_1_ can be observed: *k*_1_ varied less than 10% by variation of the nature of amines (data not shown). To simplify the demonstration of these data, Figure 4 depicts the *α* and *R*_S_ values on ZWIX(+)™ for Fmoc-Asp(O*t*Bu)-OH, Fmoc-Lys(Boc)-OH and Fmoc-Phe-OH as these results were typical for all the studied SAs. Concerning selectivity, the nature of base additives has only a slight effect on *α* (Figure 4), while for *R*_S_ TEA seemed to be the most promising base. The nature of acids exerted also some minor effect on the retention and selectivity. Based on these results, most of the experiments were carried out with mobile phases containing TEA as base and FA as acid additives.

#### 2.1.3. Effect of Counter-Ion Content

**Figure 5:**
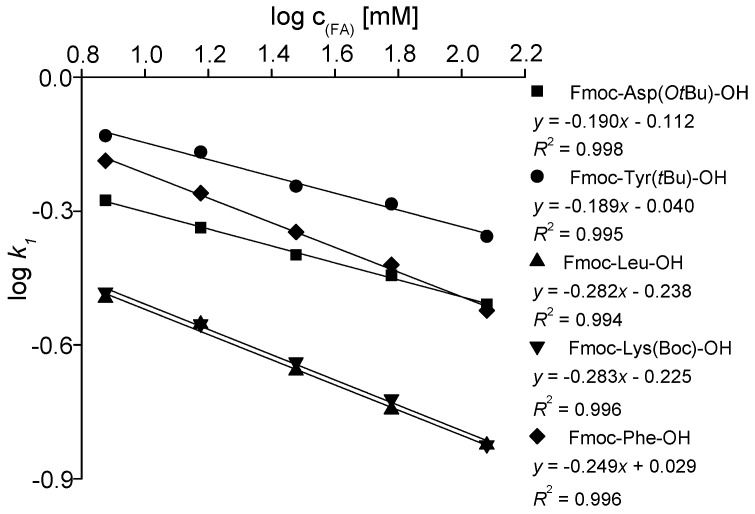
Effects of counter-ion concentration of selected analytes on retention factor, *k*_1_. Chromatographic conditions: columns, ZWIX(+)™; mobile phase, H_2_O/MeOH (1.0/99.0 *v/v*) containing TEA/FA in concentration 3.75/7.5, 7.5/15.0, 15.0/30, 30/60 and 60/120 mM/mM; flow rate, 0.6 mL·min^−1^; detection, 262 nm; ■ for Fmoc-Asp(O*t*Bu)-OH; ◆ for Fmoc-Phe-OH; ▲ for Fmoc-Leu-OH; ▼ for Fmoc-Lys(Boc)-OH; and ⚫ for Fmoc-Tyr(*t*Bu)-OH.

In the case of acidic SAs, long-range strong electrostatic (ionic) interactions between the anionic sites of the SA and the cationic site of the SO have been detected as primary ones, with additional intermolecular SO–SA interactions responsible for chiral discrimination [19,20]. The observed effects are in principle in agreement with the earlier explained simple displacement model [41,42]. Since the cationic site(s) of the SAs is blocked via the Fmoc (Boc) protection groups thus not being able taking part in the ion-pairing process, the deprotonated acid additives in the mobile phase act as counter-ions competing with the deprotonated SAs for the bonding sites of the protonated SOs. Conceptually, the concentrations of the co- and counter-ions should affect the ionic interactions, and retention can be adjusted by the addition of acidic counter-ions. Figure 5 represents the effects of concentration variations of the acidic additives on the retention on ZWIX(+)™ under HO mobile phase conditions. The application of higher counter-ion concentration resulted in lower retention in accordance with the displacement model of Kopaciewicz [41], where a linear relationship is found between log*k*_1_ and log*c_counter-ion_*. According to this model, the slopes of these plots are determined by the ratio of the effective charges of the solute and the counter-ions; the observed slopes around 0.19–0.28 correspond with the values of around 0.18–0.26 found on ZWIX(+)™ for the same SAs at SFC conditions [21] and for *β*-amino acids in PIM [39,40]. On the ZWIX(+)™, under the applied conditions, slopes differed only slightly for each enantiomer, whereas the enantioselectivity remained almost constant (data not shown).

On QN-AX™ in the mobile phase MeOH/MeCN (75/25 *v/v*) and varying the concentration of counter-ion FA between 30 and 240 mM (the acid-to-base ratio was kept at 2:1 by addition of TEA), the slopes of the log*k*_1_ vs. log*c* plots on anion-exchange columns were around 0.7 [43], which were much higher than that obtained for the zwitterionic columns. This result draws attention to the marked difference between zwitterionic SO and SOs containing only the anionic ion-exchange site. As interpretation of this finding one may have to consider also a competitive intramolecular ion-pairing effect of the zwitterionic selector motif (see Figure 2), which significantly influences the overall retention characteristics, and also the observed enantioselectivity.

#### 2.1.4. Comparison of Separation Performances of Zwitterionic and Anion-Exchanger Type CSPs in Liquid Chromatographic Mode

The separation of enantiomers of *N*^α^-Fmoc proteinogenic amino acids on ZWIX(+)™ were carried out in HO mobile phase (H_2_O/MeOH (1/99 *v/v*)) containing 30 mM TEA and 60 mM FA and on QN-AX™ in PI mobile phase (MeOH/MeCN (75/25 *v/v*)) containing 30 mM TEA and 60 mM FA. It should be noted that the same acid/base concentration level does not mean the same ionic strength because of the different activity coefficients existing in H_2_O/MeOH and MeOH/MeCN mobile phase. The registered *k_1_* values on ZWIX(+)™ were rather low (*k*_1_ ranged 0.18–0.99) (Table 1). Higher *k*_1_ values (*k*_1_ > 0.4) were obtained for polar amino acids (Fmoc-Asn-OH or Fmoc-Gln-OH) and for SAs possessing additional aromatic rings (Fmoc-Trp-OH; Pbf or Trt protecting groups). Selectivity and resolution changed parallel with *k*_1_ values. Under HO mobile phase conditions, out of the 19 *N*^α^-amino acid derivatives nine were partially and seven were baseline resolved, while Fmoc-Pro-OH, Fmoc-Thr(*t*Bu)-OH and Fmoc-Tyr(*t*Bu)-OH were not separable (Table 1).

On QN-AX™ in PIM totally different separation performances were registered. The *k*_1_ and *α* values were much higher (*k*_1_ ranged 1.4–6.8, and *α* 1.05–2.06). For most of the *N*^α^-Fmoc amino acids, *R_S_* was higher than 5.0, for Fmoc-Cys(Trt)-OH and Fmoc-Tyr(*t*Bu)-OH the R_S_ was around 2.0 and only Fmoc-Pro-OH and Fmoc-Thr(*t*Bu)-OH exhibited only partial resolution.

In summary, under liquid chromatographic conditions the zwitterionic and anion-exchanger type CSPs exhibited different separation performances for *N*^α^-Fmoc AAs. While ZWIX(+)™ exhibited in several cases insufficient separation efficiencies, on the QN-AX™ column almost all *N*^α^-Fmoc amino acids could efficiently be separated.

### 2.2. Separation of N^α^-Protected Amino Acids on Quinine-Based CSPs under SFC Conditions

To compare the LC with the SFC conditions the same four protected amino acids as model compounds were selected for the detailed investigations: Fmoc-Asp(O*t*Bu)-OH, Fmoc-Lys(Boc)-OH, Fmoc-Leu-OH and Fmoc-Phe-OH.

#### 2.2.1. Effect of Co-Solvent and Water Content

Since the elution strength of liquid CO_2_ is not enough to ensure fast elution and/or efficient separation, generally MeOH in 10–40 *v*% as co-solvent plus other polar components (water, acid and base) are needed to fulfill the requirements of ion exchange chromatography carried out in SFC mode. When applying MeOH in such a high concentration in liquid CO_2_ the mobile phases are present in subcritical rather than supercritical state. Therefore, the term “enhanced fluidity chromatography” may be more appropriate than the general term SFC.

**Figure 6:**
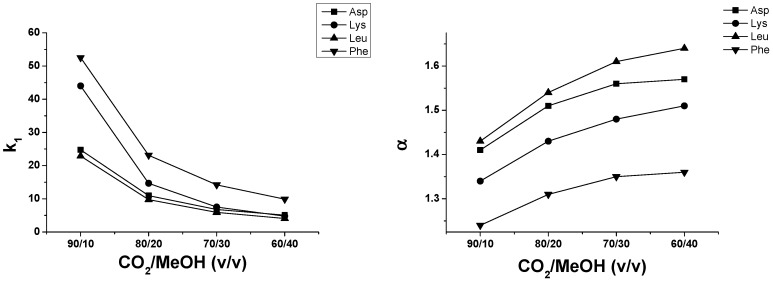

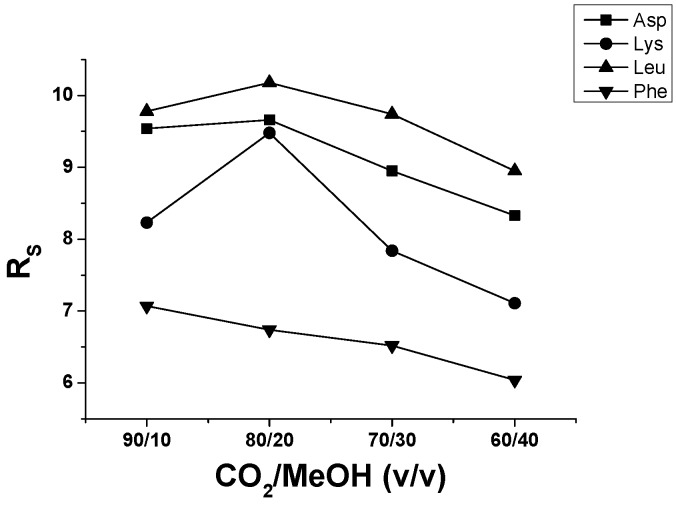
Effect of bulk solvent composition for chromatographic parameters *k*_1_, *α* and *R_S_* of selected SAs on QN-AX™ at SFC conditions. Chromatographic conditions: column, QN-AX™; mobile phase, CO_2_/MeOH (90/10, 80/20, 70/30 and 60/40 *v/v*) containing of 30.0 mM TEA and 60.0 mM FA; flow rate, 2.0 mL·min^−1^; T_col_, 40 °C; back pressure, 150 bar; detection, 262 nm. Symbols for *k*_1_, *α* and *R_S_*: ■ for Fmoc-Asp(O*t*Bu)-OH; ▼ for Fmoc-Phe-OH; ▲ for Fmoc-Leu-OH; and ⚫ for Fmoc-Lys(Boc)-OH.

As described earlier, the enantiomers of *N*^α^-Fmoc amino acids were successfully separated on *Cinchona* alkaloid-based zwitterionic ZWIX(+)™ at SFC conditions with exception of Fmoc-Pro-OH [21]. For this study the analytes were chromatographed on the anion-exchanger QN-AX™ using the same conditions. MeOH content in liquid CO_2_ was varied from 10 to 40 *v*%, while the concentrations of the additives in the mobile phase were kept constant (30 mM TEA and 60 mM FA). Figure 6 depicts results obtained for the four selected SAs: *k*_1_ values drastically decreased with increasing MeOH content, especially for Fmoc-Phe-OH and Fmoc-Lys(Boc)-OH. An increase of the MeOH content in the CO_2_ weakens strongly the electrostatic but also hydrogen bonding interactions between the SO and the SAs resulting in decreased retention. Further, reaction of MeOH with the pressurized CO_2_ at higher MeOH content significantly enhances the formation of the alkyl hydrogencarbonic acid, which may act as an additional acidic displacer (counter-ion), thus further weakening the electrostatic interaction between SAs and SO.

To promote elution of polar compounds and/or improve peak shapes, water is frequently used as modifier in SFC [44,45,46]. For investigation of the effect of water content, mobile phases composed of CO_2_/MeOH/H_2_O containing 30 mM TEA and 60 mM FA were applied. The amount of CO_2_ was constant (60 *v*%), while water was added into the MeOH phase yielding MeOH/H_2_O ratios: 40/0, 39.6/0.4, 39.2/0.8, 38/2 and 36.8/3.2 (*v/v*). Thus the total concentration of H_2_O in the eluent was varied between 0 and 3.2 *v*%. Results were very similar obtained on ZWIX(+)™ [21]. On increase of the water content, the *k*_1_, *α* and *R_S_* values of the selected Fmoc-amino acids slightly decreased (data not shown). The reaction between pressurized liquid CO_2_ and water yields carbonic acid which dissociates to hydrogencarbonate (and proton). The hydrogencarbonate can also be considered a displacer and its increasing concentration with increasing H_2_O content yields a decrease in retention.

#### 2.2.2. Effects of Acid and Base Additives

A small proportion of a polar additive in the mobile phase is frequently suggested [47,48,49,50] to tune the elution of solutes and to improve peak shapes. Moreover, additives may decrease tailing asymmetry especially during the analyses of basic compounds.

**Figure 7:**
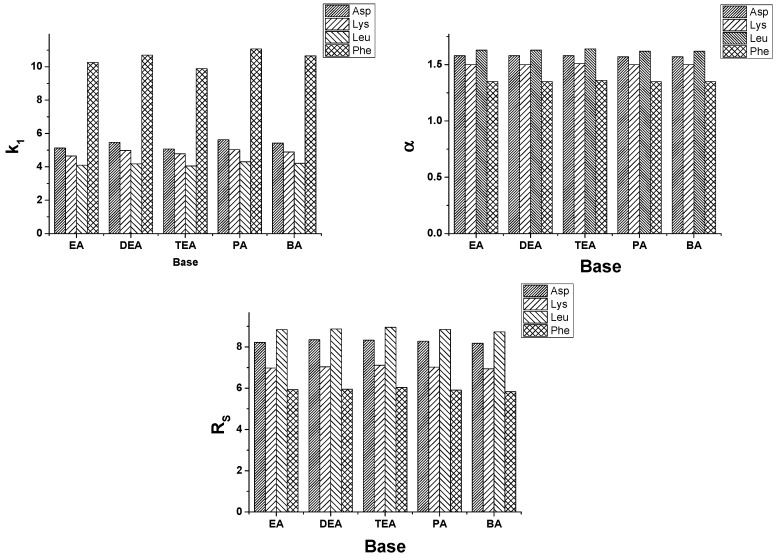
Effects of the natures of base additives on the chromatographic parameters of *k*_1_, *α* and *R_S_* of Fmoc-Asp(O*t*Bu)-OH, Fmoc-Lys(Boc)-OH, Fmoc-Leu-OH and Fmoc-Phe-OH. Chromatographic conditions: columns, QN-AX™; mobile phase, CO_2_/MeOH (60/40 *v/v*) containing 30 mM base and 60 mM FA; flow rate, 2.0 mL min^−1^; T_col_, 40 °C; back pressure, 150 bar; detection, 262 nm. Symbols: 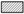 for Asp(O*t*Bu)-OH; 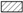 for Fmoc-Lys(Boc)-OH; 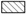 for Fmoc-Leu-OH; and 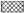 Fmoc-Phe-OH.

For this series of experiments, FA was selected as acid additive, and EA, DEA, TEA, PA and BA as base additives. In SFC mode on ZWIX(+)™, the nature of base exhibited a slight effect on the chromatographic parameters [21]. With a few exceptions, *k*_1_ decreased as the degree of alkyl substitution on the N atom increased; moreover, in most cases, *k*_1_ also decreased as the alkyl chain length on the amino group increased [21]. For the investigations of the effects of base additives on QN-AX™ under SFC conditions, experiments were carried out with CO_2_/MeOH (60/40 *v/v*) mobile phase containing 30 mM base and 60 mM FA (Figure 7). Our results demonstrated that the nature of base exhibited only very slight effect on the chromatographic parameters; e.g., *k*_1_ varied for Fmoc-Asp(O*t*Bu)-OH between 5.07 and 5.47, for Fmoc-Lys(Boc)-OH between 4.65 and 5.03, for Fmoc-Leu-OH between 4.05 and 4.30 and for Fmoc-Phe-OH between 9.89 and 11.07. Regarding *α* and *R_S_* values, the change was much smaller, within 1%–2%.

#### 2.2.3. Effects of the Counter-Ion Concentration

**Figure 8:**
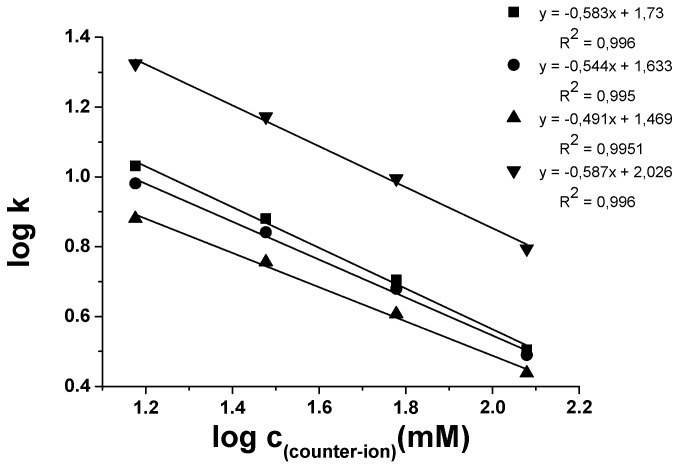
Effects of counter-ion concentration of selected analytes on retention factor, *k*_1_. Chromatographic conditions: columns, QN-AX™; mobile phase, CO_2_/MeOH (60/40 *v/v*) containing TEA/FA in concentration 7.5/15.0, 15.0/30, 30/60 and 60/120 mM/mM; flow rate, 2.0 mL·min^−1^; T_col_, 40 °C; back pressure, 150 bar; detection, 262 nm. Symbols: ■ for Fmoc-Asp(O*t*Bu)-OH; ▼ for Fmoc-Phe-OH; ▲ for Fmoc-Leu-OH; and ⚫ for Fmoc-Lys(Boc)-OH.

As described earlier on the *Cinchona* alkaloid-based zwitterionic CSPs in PI and HO mobile phases, an ion-pairing (displacement) process between the SO and the SAs dominates the retention in LC, and the displacement model also applies for the SFC mode [21]. To prove that the ion-exchange mechanism also governs retention on a genuine anion-exchanger CSP run under SFC conditions with CO_2_/MeOH as a bulk solvent, the concentrations of FA and base were varied in the range 15–120 mM, while the acid-to-base ratio (2:1) was maintained constant. Retention decreased with increasing concentration of FA plus base, and a linear relationship between log*k*_1_ and log*c_counter-ion_* for all the selected SAs was observed (Figure 8). Thus, the results obtained were in accordance with the displacement model [41,42], and it can be stated that ion-exchangers can efficiently be used in SFC mode.

For the studied QN-AX™ column, the linear relationship observed between log*k*_1_ and log*c_FA_* clearly indicate that, similar to LC conditions in SFC mode, an anion-exchange mechanism is in place. Similar to LC (mentioned before), the enantioselectivity changed only within 1% indicating that the counter-ion itself has almost no effect on the overall selectivity but it has a strong effect on the retention.

The slopes around 0.49–0.58 obtained on the QN-AX™ phase operated in SFC substantially differed from the slopes obtained on zwitterionic CSP (0.18–0.26 [21]) and are closer to the values of around 0.68–0.72 found for a QN-AX™ in LC mode [43]. The results clearly demonstrate the difference in retention mechanism on zwitterionic and anion-exchanger type CSPs whereby these differences need to be related mainly to the competing intramolecular ion-pairing effect of the SO, which is also associated with the difference of the ZWIX(+)™ and QN-AX™ selectors (see Figure 2).

#### 2.2.4. Comparison of Separation Performances of Zwitterionic and Anion-Exchanger Type CSPs Operated in SFC Mode

Table 2 provides a comprehensive set of data on the enantioseparation of a variety of *N*^α^-Fmoc-protected proteinogenic amino acids on ZWIX(+)™ and QN-AX™ in SFC mode. Despite the higher eluent strength applied (CO_2_/MeOH (60/40 *v/v*) *vs*. CO_2_/MeOH (70/30 *v/v*)), higher *k*_1_ values were detected in all cases on QN-AX™ (exception was Fmoc-Thr(*t*Bu)-OH). On both columns, higher *k*_1_ values were registered for the so-called polar amino acids like Fmoc-Asn-OH and Fmoc-Gln-OH, but also for Fmoc-amino acids containing additional large and/or aromatic protecting groups (*t*Bu, Pbf, and Trt), such as Fmoc-Arg(Pbf)-OH, Fmoc-His(Trt)-OH, Fmoc-Thr(*t*Bu)-OH, Fmoc-Cys(Trt)-OH, and Fmoc-Tyr(*t*Bu)-OH. This also applies for amino acids possessing an aromatic side-chain, i.e., Fmoc-Phe-OH and, especially, Fmoc-Trp-OH. The presence of the additional aromatic Trt group on Cys and His probably contributes to the higher retention. The highest *k*_1_ value of Fmoc-Arg(Pbf)-OH can be attributed to the presence of the additional bulky aromatic protecting group, Pbf on Fmoc-Arg. Interestingly, *k*_1_ decreases in the sequence of increasing hydrophobicity of Fmoc-Ala-OH < Fmoc-Val-OH < Fmoc-Leu-OH < Fmoc-Ile-OH. As a general trend, higher *α* and *R_S_* values were obtained on QN-AX™ than on ZWIX(+)™, but several exceptions were observed (Fmoc-Arg(Pbf)-OH, Fmoc-Trp-OH, Fmoc-Thr(*t*Bu)-OH and Fmoc-Asn-OH). Only the enantiomers of Fmoc-Pro-OH were not separable under these conditions (similarly to LC conditions).

In SFC mode both zwitterionic and anion-exchanger type CSPs are suitable for the enantioseparation of *N*^α^-Fmoc amino acids. The comparison of separation performances of liquid chromatographic and SFC mode revealed that ZWIX(+)™ possess lower separation ability in liquid chromatographic mode, while QN-AX™ can successfully be applied in both chromatographic mode.

**Figure 9:**
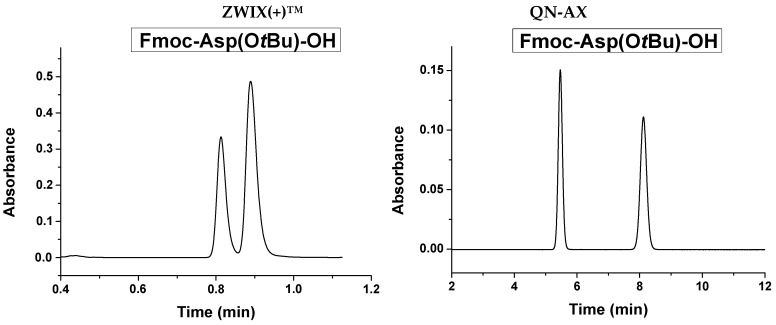

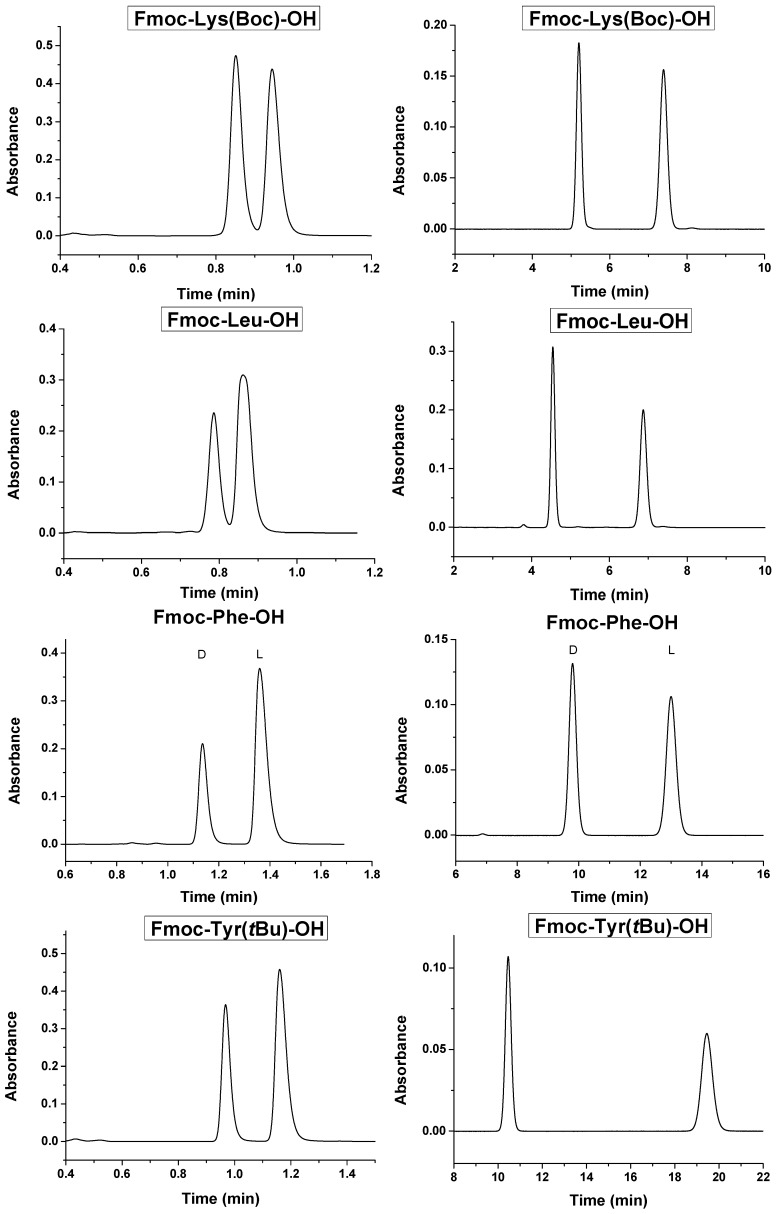
Selected chromatograms of *N^α^*-Fmoc-protected proteinogenic amino acids. Chromatographic conditions: columns, ZWIX(+)™ and QN-AX™; mobile phase, CO_2_/MeOH (60/40 *v/v*) containing 30 mM TEA and 60 mM FA; flow rate, 2.0 mL min^−1^; T_col_, 40 °C; back pressure, 150 bar; detection, 262 nm.

The *Quinine*-based columns exhibited a constant elution sequence in both chromatographic modes. D < L elution sequence was observed in all cases. Selected chromatograms are shown as examples in Figure 9.

### 2.3. Influence of Temperature on the Separation of N^α^-Fmoc Amino Acids on Quinine-Based CSPs in HO, PI and SFC Mode

The enantioseparation of selected *N*^α^-Fmoc amino acids on two columns in HO or PI and SFC mode were measured over the temperature range of 5–50 °C. The applied mobile phases for LC were H_2_O/MeOH (1/99 *v/v*) containing 3.75 mM TEA and 7.5 mM FA on ZWIX(+)^TM^ and MeOH/MeCN (75/25 *v/v*) containing 30 mM TEA and 60 mM FA for QN-AX™. For SFC we chose the conditions CO_2_/MeOH (70/30 *v/v*) containing 30 mM TEA and 60 mM FA for ZWIX(+)^TM^ and CO_2_/MeOH (60/40 *v/v*) containing 30 mM TEA and 60 mM FA for QN-AX™. Chromatographic data obtained with various temperatures are depicted in Supplementary Materials Tables S1 and S2. The *k*_1_ and *α* decreased for all cases with increasing temperature, *R_S_* in most cases also decreased, but in several cases a maximum value of *R_S_* was registered. From the chromatographic data, van’t Hoff plots were constructed and the ln*α* vs. *1/T* curves gave linear plots as indicated by the correlation coefficient in the Table 3.

According to the data summarized in Table 3, the *Δ*(*ΔH*°) values were all negative (ranged from −0.8 to −7.5 kJ·mol^−1^) indicating a favorable exothermic process in the course of transfer of analyte from the mobile to the stationary phase. It was generally observed that *Δ*(*ΔH*°) values were more negative on both ZWIX(+)™ and QN-AX™ under liquid chromatographic conditions than under SFC conditions (the only exception was Fmoc-Leu-OH).

Under the applied conditions, *Δ*(*ΔS*°) ranged from −1.1 to −23.7 J·mol^−1^·K^−1^ (Table 3). In most cases, under LC conditions, the *Δ*(*ΔS*°) values on ZWIX(+)™ were more negative, while under SFC conditions on QN-AX™ more negative *Δ*(*ΔS*°) values were registered.

The most negative *Δ*(*ΔG*°) values (calculated at 298 K) were generally obtained on QN-AX™ working either in liquid chromatographic or SFC mode.

The relative contribution to the free energy of adsorption can be represented through the calculation of the enthalpy/entropy ratio *Q* [*Q* = *Δ*(*ΔH*°)/[298 × *Δ*(*ΔS*°)]. As indicated in Table 3, the enantioselective discrimination was enthalpically driven (*Q* > 1.0) in all cases and with few exceptions the highest *Q* values were most often obtained with QN-AX™.

## 3. Materials and Methods

### 3.1. Chemicals and Reagents

Besides the *N^α^*-Fmoc protection, the other reactive sites of proteinogenic amino acids were also protected to make them appropriate for peptide synthesis protocols: *t*-butyloxycarbonyl (Boc) for Lys, *t*-butyl (*t*Bu) for Ser, Thr and Tyr, *O*-*t*-butyl (O*t*Bu) for Asp and Glu, triphenylmethyl (trityl, Trt) for Cys and His, and *N^ω^*-2,2,4,6,7-pentamethyldihydrobenzofuran-5-sulfonyl (Pbf) for Arg (Figure 1). The protected amino acid derivatives were obtained from different sources. l-amino acids **1** and **2** were purchased from Reanal (Budapest, Hungary), **3**–**14** and **16** from Orpegen Pharma Gmbh (Heidelberg, Germany), **15** from GL Biochem (Shanghai) Ltd (Shanghai, China), **17** from Iris Biotech Gmbh (Marktredwitz, Germany) and **18** from Merck (Darmstadt, Germany). d-amino acids **3**, **4**, **6**, **9**, **10**, **12**–**14**, **16**, **18** and **19** were obtained from Bachem AG (Bubendorf, Switzerland), **1**, **2**, **7**, **8**, **11**, **15** and **17** from AK Scientific, Inc. (Union City, CA, USA) and **5** from Advanced ChemTech (Louisville, KY, USA).

MeOH and MeCN of HPLC grade were provided by VWR International (Radnor, PA, USA). The base additives were ethylamine (EA), diethylamine (DEA), triethylamine (TEA), propylamine (PA) and butylamine (BA), the acid additives were formic acid (FA) and acetic acid (AcOH), all obtained from VWR International. Liquid CO_2_ was from Messer (Budapest, Hungary). The ultrapure water was obtained from the Ultrapure Water System, Puranity TU UV/UF (VWR International bvba, Leuven, Belgium).

### 3.2. Apparatus and Chromatography

Liquid chromatography was performed on two chromatographic systems. Waters Breeze system consisted of a 1525 binary pump, a 487 dual-channel absorbance detector, a 717 plus autosampler and Empower 2 data manager software (Waters Chromatography, Milford, MA, USA). A Lauda Alpha RA8 thermostat (Lauda Dr. R. Wobser Gmbh, Lauda-Königshofen, Germany) was applied for the thermostation of columns.

1100 Series Agilent system consisted of a solvent degasser, a pump, an autosampler, a column thermostat, a multiwavelength UV-Vis detector (Agilent Technologies, Waldbronn, Germany) and a Corona-charged aerosol detector from ESA Biosciences, Inc. (Chelmsford, MA, USA). Data acquisition and analysis were carried out with ChemStation chromatographic data software from Agilent Technologies.

Stock solutions of amino acids (1 mg·mL^−1^) were prepared by dissolving them in methanol. For determination of the dead-times (*t*_0_) of the columns, a methanolic solution of acetone was applied at each investigated temperature and eluent composition. The flow rate was 0.6 mL·min^−1^ and the column temperature was 25 °C if not otherwise stated.

For subcritical fluid chromatography Waters Acquity Ultra Performance Convergence Chromatography^TM^ (UPC^2^, Waters Chromatography, Milford, MA, USA) system was applied with a binary solvent delivery pump, an autosampler including the volume injection system, a backpressure regulator, a column oven and a PDA detector. For system control and data acquisition Empower 2 software (Waters Chromatography) was used.

SFC was performed in isocratic mode at a flow rate of 2.0 mL·min^−1^ and a column temperature of 40 °C (if not otherwise stated) and the outlet pressure was maintained at 150 bar for all columns. The mobile phase consisted of CO_2_ and MeOH in different ratios (*v/v*) containing different additives (H_2_O, acids and bases).

In SFC mode, the dead-time of the columns (*t_0_*) was determined at a first negative signal by injecting MeOH. All SAs were dissolved in MeOH in the concentration range 0.5–1.0 mg·mL^−1^. In both chromatographic systems, SAs were detected by UV absorption at 262 nm.

The Chiralpak ZWIX(+)™ and QN-AX™ columns (150 mm × 3.0 mm I.D., 3-μm particle size) (Figure 2) were from Chiral Technologies Europe (Illkirch, France).

## 4. Conclusions

A comparative study of the resolution of the enantiomers of nineteen *N*^α^-Fmoc amino acids on *quinine*-based zwitterionic and anion exchanger-type CSPs under hydro-organic, polar-ionic and SFC conditions was carried out. Under all the chromatographic conditions applied in this study, retention was found to be primarily based on ionic interactions, whereas additional intermolecular interactions were responsible for chiral discrimination.

Under LC conditions, the zwitterionic CSP exhibited limited enantioseparation performances, however, on the anion-exchanger type CSP, almost all *N*^α^-Fmoc amino acids could efficiently be resolved under liquid phase conditions. The nature of base and acid added to the eluent systems had only a slight effect on retention and enantioselectivity, while the effect of the counter-ion could be described by the stoichiometric displacement model.

Under SFC conditions, retention strongly depended on the MeOH content of the mobile phase; an increased MeOH content resulted in reduced retention times. Thus, the SFC conditions became similar to polar ionic combinations. In SFC, similar to the results obtained under LC conditions, the nature of base and acid additives had only moderate effect on the chromatographic behavior. The observed linear relationship between log*k*_1_ and log*c_FA_* strongly supports the existence of the ion-exchange mechanism for the studied anion-exchanger type column also in SFC. Both zwitterionic and anion-exchanger type CSPs were found to be suitable for the enantioseparation of *N^α^*-Fmoc amino acids in LC and SFC mode.

Elution sequence was determined for all the studied analytes, where a general rule could be observed: D enantiomers eluted before the L ones both in liquid and SFC mode. Based on the results of the thermodynamic calculations, the enantioselective discrimination was found to be enthalpically driven for all the studied analytes.

## Figures and Tables

**Table 1:** Chromatographic data, retention factor (*k*), selectivity factor (*α*), resolution (*R_S_*) and elution sequence of *N*-Fmoc-protein amino acids on ZWIX(+)™ and QN-AX CSPs at liquid chromatographic condition.

Compound	Column	*k*_1_	*α*	*R_S_*	Elution Sequence
Fmoc-Asp(O*t*Bu)-OH	ZWIX(+)™	0.36	1.32	1.16	*D < L*
	QN-AX™	2.38	1.82	8.77	*D < L*
Fmoc-Glu(O*t*Bu)-OH	ZWIX(+)™	0.22	1.32	0.64	*D < L*
	QN-AX™	1.97	1.88	9.54	*D < L*
Fmoc-Lys(Boc)-OH	ZWIX(+)™	0.19	1.43	1.01	*D < L*
	QN-AX™	1.32	1.80	6.20	*D < L*
Fmoc-Arg(Pbf)-OH	ZWIX(+)™	0.99	1.91	4.67	*D < L*
	QN-AX™	2.74	1.59	7.87	*D < L*
Fmoc-His(Trt)-OH	ZWIX(+)™	0.63	1.66	2.89	*D < L*
	QN-AX™	1.85	1.99	10.49	*D < L*
Fmoc-Ala-OH	ZWIX(+)™	0.25	1.36	0.88	*D < L*
	QN-AX™	2.18	1.50	6.67	*D < L*
Fmoc-Val-OH	ZWIX(+)™	0.20	1.54	0.77	*D < L*
	QN-AX™	1.88	2.06	12.66	*D < L*
Fmoc-Leu-OH	ZWIX(+)™	0.18	1.36	0.70	*D < L*
	QN-AX™	1.58	1.83	8.53	*D < L*
Fmoc-Ile-OH	ZWIX(+)™	0.18	1.43	0.69	*D < L*
	QN-AX™	1.80	2.05	10.62	*D < L*
Fmoc-Phe-OH	ZWIX(+)™	0.38	1.63	2.11	*D < L*
	QN-AX™	3.26	1.55	7.74	*D < L*
Fmoc-Trp-OH	ZWIX(+)™	0.80	2.36	6.82	*D < L*
	QN-AX™	4.11	1.64	8.70	*D < L*
Fmoc-Met-OH	ZWIX(+)™	0.33	1.41	1.31	*D < L*
	QN-AX™	2.84	1.67	9.83	*D < L*
Fmoc-Pro-OH	ZWIX(+)™	0.27	1.00	0.00	*-*
	QN-AX™	1.88	1.06	1.06	*D < L*
Fmoc-Ser(*t*Bu)-OH	ZWIX(+)™	0.22	1.41	0.87	*D < L*
	QN-AX™	2.14	1.35	5.42	*D < L*
Fmoc-Thr(*t*Bu)-OH	ZWIX(+)™	0.19	1.00	0.00	*-*
	QN-AX™	1.72	1.05	0.52	*-*
Fmoc-Cys(Trt)-OH	ZWIX(+)™	0.59	1.49	2.36	*D < L*
	QN-AX™	6.77	1.14	2.68	*D < L*
Fmoc-Tyr(*t*Bu)-OH	ZWIX(+)™	0.52	1.00	0.00	*-*
	QN-AX™	3.89	1.17	2.11	*D < L*
Fmoc-Asn-OH	ZWIX(+)™	0.73	2.06	5.50	*D < L*
	QN-AX™	3.45	1.33	5.47	*D < L*
Fmoc-Gln-OH	ZWIX(+)™	0.45	1.54	2.11	*D < L*
	QN-AX™	2.75	1.98	10.61	*D < L*

Chromatographic conditions: column, ZWIX(+)™ and QN-AX™; mobile phase, on ZWIX(+)™ H_2_O/MeOH (1/99 *v/v*) containing 30 mM TEA and 60 mM FA and on QN-AX™ MeOH/MeCN (75/25 *v/v*) containing 30 mM TEA and 60 mM FA; flow rate, 0.6 mL·min^−1^; detection, 262 nm; temperature, ambient.

**Table 2:** Chromatographic data, retention factor (*k*), selectivity factor (*α*), resolution (*R_S_*) and elution sequence of *N*-Fmoc-protein amino acids on ZWIX(+)™ and QN-AX™ CSPs at SFC condition.

Compound	Column	*k*_1_	*α*	*R_S_*	Elution Sequence
Fmoc-Asp(O*t*Bu)-OH	ZWIX(+)™ *	1.76	1.18	2.43	*D < L*
	QN-AX™	5.074	1.58	8.33	*D < L*
Fmoc-Glu(O*t*Bu)-OH	ZWIX(+)™ *	1.66	1.17	2.13	*D < L*
	QN-AX™	5.01	1.64	8.86	*D < L*
Fmoc-Lys(Boc)-OH	ZWIX(+)™ *	2.22	1.21	2.63	*D < L*
	QN-AX™	4.781	1.51	7.11	*D < L*
Fmoc-Arg(Pbf)-OH	ZWIX(+)™ *	24.39	1.65	9.95	*D < L*
	QN-AX™	27.50	1.33	5.26	*D < L*
Fmoc-His(Trt)-OH	ZWIX(+)™ *	5.19	1.31	4.36	*D < L*
	QN-AX	8.20	1.47	4.64	*D < L*
Fmoc-Ala-OH	ZWIX(+)™ *	2.20	1.15	2.03	*D < L*
	QN-AX	5.86	1.40	6.49	*D < L*
Fmoc-Val-OH	ZWIX(+)™ *	1.60	1.24	2.79	*D < L*
	QN-AX	4.46	1.71	9.87	*D < L*
Fmoc-Leu-OH	ZWIX(+)™ *	1.62	1.20	2.11	*D < L*
	QN-AX™	4.05	1.64	8.95	*D < L*
Fmoc-Ile-OH	ZWIX(+)™ *	1.42	1.21	1.49	*D < L*
	QN-AX™	4.20	1.72	9.82	*D < L*
Fmoc-Phe-OH	ZWIX(+)™ *	3.35	1.31	4.53	*D < L*
	QN-AX™	9.887	1.36	6.04	*D < L*
Fmoc-Trp-OH	ZWIX(+)™ *	16.40	2.02	10.70	*D < L*
	QN-AX	26.98	1.45	7.42	*D < L*
Fmoc-Met-OH	ZWIX(+)™ *	2.95	1.23	3.41	*D < L*
	QN-AX™	8.55	1.52	8.13	*D < L*
Fmoc-Pro-OH	ZWIX(+)™ *	1.66	1.00	0.00	*-*
	QN-AX™	4.04	1.00	0.00	*-*
Fmoc-Ser(*t*Bu)-OH	ZWIX(+)™ *	1.26	1.13	0.96	*D < L*
	QN-AX™	3.98	1.23	3.64	*D < L*
Fmoc-Thr(*t*Bu)-OH	ZWIX(+)™ *	3.67	1.95	9.42	*-*
	QN-AX™	2.73	1.17	2.70	*D < L*
Fmoc-Cys(Trt)-OH	ZWIX(+)™ *	7.92	1.07	1.20	*D < L*
	QN-AX™	15.84	1.73	10.49	*D < L*
Fmoc-Tyr(*t*Bu)-OH	ZWIX(+)™ *	2.67	1.33	4.39	*-*
	QN-AX™	10.63	1.94	12.36	*D < L*
Fmoc-Asn-OH	ZWIX(+)™ *	8.95	1.35	5.73	*D < L*
	QN-AX™	14.54	1.23	3.94	*D < L*
Fmoc-Gln-OH	ZWIX(+)™ *	6.57	1.49	7.41	*D < L*
	QN-AX™	13.04	2.31	15.10	*D < L*

Chromatographic conditions: column, ZWIX(+)™ and QN-AX; mobile phase, on ZWIX(+)™ CO_2_/MeOH (70/30 *v/v*) containing 30 mM TEA and 60 mM FA and on QN-AX™ CO_2_/MeOH (60/40 *v*/*v*) containing 30 mM TEA and 60 mM FA; flow rate, 2.0 mL min^−1^; detection, 262 nm; T_col_, 40 °C; back pressure, 150 bar; *, data from Ref. [21].

**Table 3:** Thermodynamic parameters, *Δ(ΔH°),* *Δ(ΔS°),* *T*x*Δ*(*ΔS°*), *Δ(ΔG°),* and *Q* values of *N*-Fmoc-protected protein amino acids on ZWIX(+)^TM^ and QN-AX™ CSP in liquid chromatographic and SFC mode.

Compound	Column/Mobile Phase	−Δ(Δ*H°*) (kJ·mol^−1^)	−Δ(Δ*S°*) (J·mol^−1^·K^−1^)	−TxΔ(Δ*S°*)*_298 K_* (kJ·mol^−1^)	−Δ(Δ*G°*)_298 K_ (kJ·mol^−1^)	*Q*
Fmoc-Asp(O*t*Bu)-OH	ZWIX(+)™ HO/k	5.6	16.7	5.0	0.6	1.1
	QN-AX™PIM/w	5.5	13.5	4.0	1.5	1.4
	ZWIX(+)™ ∗ SFC/y	0.8	1.3	0.4	0.4	2.0
	QN-AX™SFC/x	3.6	7.5	2.2	1.4	1.6
Fmoc-Lys(Boc)-OH	ZWIX(+)™HO/k	3.5	10.1	3.0	0.5	1.2
	QN-AX ™PIM/w	3.8	7.8	2.3	1.5	1.7
	ZWIX(+)™ ∗ SFC/y	1.4	2.7	0.8	0.6	1.8
	QN-AX ™SFC/x	1.9	2.5	0.7	1.2	2.7
Fmoc-Leu-OH	ZWIX(+)™HO/k	2.8	7.0	2.1	0.7	1.2
	QN-AX ™ PIM/w	4.2	9.1	2.7	1.5	1.6
	ZWIX(+)™ ∗ SFC/y	0.8	1.1	0.3	0.5	2.7
	QN-AX ™ SFC/x	5.1	12.1	3.6	1.5	1.4
Fmoc-Phe-OH	ZWIX(+)™ HO/k	7.1	19.7	5.9	1.2	1.2
	QN-AX ™ PIM/w	4.0	9.6	2.9	1.1	1.4
	ZWIX(+)™ ∗ SFC/y	2.0	4.2	1.3	0.7	1.5
	QN-AX ™ SFC/x	2.1	4.3	1.3	0.8	1.6
Fmoc-Tyr(*t*Bu)-OH	ZWIX(+)™ HO/k	7.5	23.7	7.1	0.4	1.1
	QN-AX ™ PIM/w	2.6	7.3	2.2	0.4	1.2
	ZWIX(+)™ ∗ SFC/y	2.7	6.2	1.8	0.9	1.5
	QN-AX ™ SFC/x	2.4	2.4	0.7	1.7	3.4

Chromatographic conditions: column, ZWIX(+)^TM^ and QN-AX™; mobile phase, k, H_2_O/MeOH (1/99 *v/v*) containing 3.75 mM TEA and 7.5 mM FA, w, MeOH/MeCN (75/25 *v/v*) containing 30 mM TEA and 60 mM FA, y, CO_2_/MeOH (70/30 *v/v*) containing 30 mM TEA and 60 mM FA, x, CO_2_/MeOH (60/40 *v/v*) containig 30 mM TEA and 60 mM FA; flow rate, 0.6 mL·min^−1^ or 2 mL·min^−1^ (SFC); detection, 262 nm; *R^2^*, correlation coefficient of van’t Hoff plot, ln*α* vs. 1/*T* curves; *Q* = Δ(Δ*H*°)/(298 × Δ(Δ*S*°)); ∗, data from Ref. [21].

## Supplementary Materials

The followings are available online http://www.mdpi.com/1420-3049/21/11/1579/s1.
