# Supplementary material for: A Comparative Study of Enantioseparations of Nα-Fmoc Proteinogenic Amino Acids on Quinine-Based Zwitterionic and Anion Exchanger-Type Chiral Stationary Phases under Hydro-Organic Liquid and Subcritical Fluid Chromatographic Conditions
Source: Molecules. 2016 Nov 22;21(11):1579. doi: 10.3390/molecules21111579 (PMC6273653; doi:10.3390/molecules21111579)
Supplement: Supplementary file 1 [file molecules-21-01579-s001.pdf]

Supplementary Materials: A Comparative Study of Enantioseparations of N$^{\alpha}$-Fmoc Proteinogenic Amino Acids on *Quinine*-Based Zwitterionic and Anion Exchanger-Type Chiral Stationary Phases under Hydro-Organic Liquid and Subcritical Fluid Chromatographic Conditions

Gyula Lajkó, Nóra Grecsó, Gábor Tóth, Ferenc Fülöp, Wolfgang Lindner, Antal Péter and István Ilisz

Table S1. Temperature dependence of retention factor of first eluting enantiomer (k_1), separation factor (α) and resolution (R_S) of *N*-Fmoc-protected protein amino acids on ZWIX(+)™ and QN-AX™ under liquid chromatographic conditions.

Compound	Column	Eluent	k_1, α, R_S	Temperature (°C)				
				5	10	20	30	40
Fmoc-Asp(O*t*Bu)-OH	ZWIX(+)™	**k**	k_1	0.46	0.45	0.44	0.41	0.37
			α	1.50	1.40	1.32	1.22	1.14
			R_S	1.30	1.04	1.04	1.08	0.91
	QN-AX™	**w**	k_1	3.27	2.67	2.57	2.36	2.16
			α	2.15	2.03	1.89	1.76	1.63
			R_S	11.50	11.33	10.88	10.39	9.25
Fmoc-Lys(Boc)-OH	ZWIX(+)™	**k**	k_1	0.30	0.30	0.30	0.29	0.26
			α	1.37	1.33	1.27	1.21	1.15
			R_S	1.03	0.93	0.94	0.65	0.76
	QN-AX™	**w**	k_1	1.85	1.69	1.48	1.35	1.25
			α	1.99	1.95	1.86	1.76	1.66
			R_S	10.11	10.95	10.53	7.29	7.91
Fmoc-Leu-OH	ZWIX(+)™	**k**	k_1	0.30	0.30	0.30	0.29	0.26
			α	1.47	1.43	1.38	1.33	1.27
			R_S	0.57	0.53	0.27	0.44	0.20
	QN-AX™	**w**	k_1	2.04	1.85	1.63	1.55	1.41
			α	2.06	1.98	1.88	1.77	1.68
			R_S	9.33	9.25	10.00	7.62	7.57
Fmoc-Phe-OH	ZWIX(+)™	**k**	k_1	0.61	0.59	0.55	0.53	0.49
			α	2.02	1.90	1.73	1.55	1.43
			R_S	3.15	3.06	2.62	1.75	2.05
	QN-AX™	**w**	k_1	5.03	3.54	3.44	3.20	2.88
			α	1.75	1.72	1.60	1.53	1.45
			R_S	8.76	9.29	8.85	8.16	7.33
Fmoc-Tyr(*t*Bu)-OH	ZWIX(+)™	**k**	k_1	0.77	0.74	0.66	0.59	0.57
			α	1.47	1.38	1.26	1.12	1.02
			R_S	1.49	1.21	1.06	0.20	0.20
	QN-AX™	**w**	k_1	5.33	4.21	4.06	3.68	3.23
			α	1.27	1.25	1.20	1.16	1.12
			R_S	3.87	3.58	3.31	2.75	2.18

Chromatographic conditions: column, ZWIX(+)™ and QN-AX™; mobile phase, **k**, H_2O/MeOH (1/99 *v*/*v*) containing 3,75 mM TEA and 7,5 mM FA, **w**, MeOH/MeCN (75/25 *v*/*v*) containing 30 mM TEA and 60 mM FA; flow rate, 0.6 mL min^{-1}; detection, 262 nm.

Table S2. Effect of temperature on chromatographic parameter k_1, α, and R_S of *N*-Fmoc-protected amino acids on QN-AX™ column under SFC conditions.

**Compound**	**k_1, α, R_S**	**Temperature (°C)**			
		**20**	**30**	**40**	**50**
**Fmoc-Asp-(O*t*Bu)-OH**	k_1	5.83	5.41	5.06	4.68
	α	1.72	1.67	1.59	1.50
	R_S	8.61	8.59	8.03	7.36
**Fmoc-Lys(Boc)-OH**	k_1	5.65	5.35	5.06	4.78
	α	1.59	1.57	1.51	1.49
	R_S	7.17	7.19	6.83	6.31
**Fmoc-Leu-OH**	k_1	-	4.94	4.57	4.14
	α	-	1.73	1.62	1.53
	R_S	-	9.21	8.76	8.02
**Fmoc-Phe-OH**	k_1	12.07	11.12	10.16	9.03
	α	1.39	1.37	1.33	1.29
	R_S	5.99	5.76	5.58	5.19
**Fmoc-Tyr(*t*Bu)-OH**	k_1	12.21	12.00	10.99	9.17
	α	2.00	1.96	1.89	1.83
	R_S	10.98	11.25	11.88	12.32

Chromatographic conditions: Column, Chiralpak QN-AX™; mobile phase, CO_2/MeOH (60/40 *v*/*v*) containing 30 mM TEA and 60 mM FA; flow rate, 2 mL min^{-1}; detection, 264 nm; T_{col}, 20–40 °C; back pressure, 150 bar
